# Influence of the energy concentration and the standardized ileal digestible lysine content of the diet on performance and egg quality of brown-egg laying hens from 18 to 41 weeks of age

**DOI:** 10.1016/j.psj.2022.102197

**Published:** 2022-09-21

**Authors:** R. Scappaticcio, L. Cámara, J. Herrera, G.G. Mateos, A.F. de Juan, G. Fondevila

**Affiliations:** ⁎ Camar Agroalimentaria S.L., Toledo, Spain; † Departamento de Producción Agraria, Universidad Politécnica de Madrid, 28040 Madrid, Spain

**Keywords:** digestible lysine, egg quality, energy concentration, laying hen production

## Abstract

The influence of the energy and the standardized ileal digestible lysine (**DLys**) content of the diet on egg production and egg quality, was studied in brown-egg laying hens from 18 to 41 wk of age. The experimental design was completely randomized with 10 treatments organized as a 2 × 5 factorial with 2 energy concentrations (2,750 and 2,800 kcal AMEn/kg) and 5 levels of DLys (values varied from 0.66 to 0.78% and 0.67 to 0.79%, for the low and high energy diets, respectively). Each treatment was replicated 10 times (10 hens per replicate). The data were analyzed using the MIXED procedure of SAS with energy concentration and DLys content of the diets as main effects. In addition, the effects of the DLys on the variables studied were partitioned into its lineal and quadratic components. From 18 to 21 wk of age (pre-peak phase), diet composition had limited effects on egg production. From 22 to 41 wk of age (peak phase), however, an increase of 50 kcal AMEn/kg diet increased egg weight (*P* < 0.05) and tended to improve energy intake (*P* = 0.083) and feed conversion ratio (*P* = 0.074). An increase in DLys improved linearly (*P* < 0.001) egg production, egg weight, egg mass, feed conversion, and energy conversion ratio, and tended to increase BW gain (*P* = 0.074). Diet composition did not any affect egg quality trait except shell strength that increased linearly (*P* < 0.05) with increases in the DLys. Cumulatively (18–41 wk of age), egg weight increased (*P* < 0.05) as the energy and the DLys content of the diet increased. In summary, an increase in energy and DLys content of the diet had limited effects on egg production during the prepeak phase but improved egg production, feed conversion ratio, and BW gain during the peak phase. The data indicate that hens require at least 839 mg DLys/d to maximize egg production in the peak production phase.

## INTRODUCTION

Metabolizable energy and standardized ileal digestible Lys (**DLys**) content of the diets, are two key factors affecting feed cost and egg production in commercial layer operations. Hens adjust feed intake (**FI**) to satisfy their energy requirements for maintenance, BW gain, and egg mass production (Grobas et al., 1999; Wu et al, 2005; dePersio et al., 2015). Brown layers are lighter, produce more eggs, and consume less feed than 3 decades ago (Joly, 2012; Scappaticcio et al., 2021). Consequently, the implementation of an adequate feeding strategy in the prepeak phase, is critical to optimize production during the whole egg production cycle.

Under stressful situations, such as hot weather or poor management practices, young hens fed low energy diets, might not be able to maintain an adequate energy and nutrients intakes to satisfy their requirements for BW gain and the development of the skeleton and the organs of the reproductive system. As a consequence, highly concentrated diets have been recommended to allow a rapid increase in BW and egg mass production (DeGroote, 1972; Wu et al., 2005; Pérez-Bonilla et al., 2012b). However, high energy diets are costly and might reduce energy efficiency because part of the energy consumed is derived to body fat deposition rather than to an increase in egg mass production (Pérez-Bonilla et al., 2012a).

The requirements in DLys of laying hens have been studied by numerous authors with extremely variable recommendations (Faria et al., 2003; Bregendahl et al., 2008; Lemme, 2009; Kumar et al., 2018; Scappaticcio et al., 2021). Factors, such as strain, age, BW, egg mass production, management practices, ambient temperature, and health status, affect FI of the hens and thus, the percentage of amino acids (**AA**) required in the diets (Cupertino et al., 2009; Silva et al., 2015; Herrera et al., 2018). Consequently, the relative importance of energy and DLys content of the diet on egg production, might vary depending on the age period studied. In this respect, most research conducted to estimate the requirements in essential AA of laying hens during the peak production phase, started with hens with at least 22 to 25 wk of age (Schutte and Smink, 1998; Bregendahl et al., 2008; Rocha et al., 2009; Spangler et al., 2018). However, under commercial conditions, young hens are often fed a common diet from 2 wk after the arrival to the laying barn (16–17 wk of age and at around 10% egg production) until peak production, a period in which FI and the requirements of the hens in DLys differ widely.

In general, the AA requirements of poultry are formulated based on the ideal protein concept (Baker, 1997; Bregendahl et al., 2008; FEDNA, 2018; Lohmann, 2020) with Lys, the second limiting AA in most diets for laying hens, as a reference. In practice, the DLys content of the diet is adjusted to ensure a correct balance between AA and energy, taking into consideration the genetic improvement that has occurred for the last decade (Macelline et al., 2021). The objective of this research was to determine the effects of increasing the energy concentration of the diet from 2,750 to 2,800 Kcal AMEn/kg and the DLys content from 0.665 to 0.785% while maintaining constant the DLys:AMEn ratio, on egg production and egg quality traits of brown-egg laying hens during the prepeak (18–21 wk of age) and peak production (22–41 wk of age) phases.

## MATERIALS AND METHODS

### Husbandry, Diets, and Experiment Design

The procedures used in this research were approved by the Animal Ethics Committee of the Universidad Politécnica de Madrid and were in compliance with the Spanish Guidelines for the care and use of animals in research (Boletín Oficial Del Estado, 2013). In total, 1,000 Lohmann Brown Classic hens were selected at random from a commercial flock at 17 wk of age and housed in the second floor of a cage battery system within an environmentally controlled commercial barn (110,000 birds). At 18 wk of age, the hens were weighed individually and allotted in groups of 10 to 100 adjacent cages, with similar average BW. The enriched cages (Facco S.p.A., Padova, Italy) measured 120 × 63 × 45 cm (width × depth × height) and were equipped with an open trough feeder and 2 low pressure nipple drinkers. Barn temperature was recorded daily, with a maximum of 26 ± 2°C (July, second period of the experiment) and a minimum of 23 ± 4°C (November, last period of the experiment). The lighting program consisted in increasing the light period of the pullets, starting at 16 wk of age, to reach 16 h at 21 wk and then, the program was maintained constant until the end of the experiment. Birds had free access to water and feed in mash form throughout the experiment.

The experimental design was completely randomized with 10 diets arranged as a 2 × 5 factorial with two energy concentrations (2,750 vs. 2,800 Kcal AMEn/kg) and 5 DLys to AMEn ratios (0.24–0.28 mg DLys/kcal AMEn). Consequently, the DLys content of the two set of diets (low and high AMEn diets) varied slightly (from 0.66 to 0.78% and from 0.67 to 0.79%, respectively). All other indispensable AA were formulated according to the ideal protein concept. To ensure that DLys was the AA limiting egg production in all cases, the experimental diets were formulated to exceed by at least 2% units, the desired ratio between key indispensable AA and DLys content. As a result, compared to FEDNA (2018) recommendations, the calculated ratio between the indispensable AA and DLys of the experimental diets was 142 versus 104% for Arg, 82 versus 80% for Ile, 72 versus 70% for Thr, 23 versus 21% for Trp, 90 versus 88% for TSAA, and 96 versus 89% for Val. Before feed manufacturing, the CP, ether extract, and AA content of the 3 main ingredients (corn, soybean meal, and sunflower meal) used and the gross energy and the linoleic acid content of the soy soapstocks were analyzed to ensure that their chemical composition were close to values reported by the FEDNA (2021) tables. For the manufacturing of the experimental feeds, the 2 extreme diets of each set (high and low AMEn content) were formulated and the intermediate diets resulted from the mixing of the 2 extreme diets in adequate proportions. The ingredient composition and the calculated and determined nutrient content of the experimental diets are presented in Table 1.

**Table 1:** Ingredient composition and chemical analyses of the experimental diets (% as fed basis).

	2,750 kcal AMEn/kg					2,800 kcal AMEn/kg				
DLys^1^ (%)	0.66	0.69	0.72	0.75	0.78	0.67	0.70	0.73	0.76	0.79
Ingredient										
Corn	59.13	58.12	57.10	56.10	55.10	56.67	55.57	54.49	53.44	52.35
Soybean meal (47% CP)	10.77	12.91	15.05	17.19	19.33	11.59	13.77	15.95	18.12	20.30
Sunflower meal (36% CP)	16.14	15.00	13.87	12.73	11.59	16.11	15.00	13.89	12.78	11.67
Soy oil soapstocks	3.52	3.53	3.54	3.55	3.56	4.92	4.94	4.96	4.98	5.00
Calcium carbonate^2^	8.96	8.96	8.96	8.96	8.96	9.19	9.19	9.19	9.18	9.18
Dicalcium phosphate	0.67	0.66	0.66	0.65	0.64	0.71	0.70	0.70	0.68	0.68
Sodium chloride	0.35	0.35	0.35	0.35	0.35	0.35	0.35	0.35	0.35	0.35
L-Lysine-HCl (78%)	0.15	0.14	0.12	0.11	0.09	0.14	0.13	0.11	0.10	0.08
DL-Methionine (99%)	0.11	0.13	0.15	0.16	0.18	0.12	0.15	0.16	0.17	0.19
Premix^3^	0.20	0.20	0.20	0.20	0.20	0.20	0.20	0.20	0.20	0.20
Determined analysis										
Dry matter	88.5	88.6	88.8	89.0	89.1	88.7	88.8	89.0	88.9	89.2
Gross energy (kcal/kg)	3,786	3,802	3,814	3,824	3,832	3,951	3,973	4,004	4,082	4,108
Crude protein	16.0	16.2	16.7	17.2	17.5	16.1	16.3	16.7	17.3	17.7
Amino acid										
Arg	1.07	1.09	1.10	1.15	1.16	1.08	1.14	1.14	1.18	1.20
Lys	0.76	0.81	0.83	0.85	0.87	0.78	0.82	0.85	0.88	0.91
Met	0.42	0.44	0.45	0.48	0.51	0.43	0.44	0.46	0.48	0.51
Met + Cys	0.68	0.72	0.73	0.75	0.80	0.70	0.72	0.76	0.78	0.83
Thr	0.57	0.58	0.60	0.63	0.67	0.58	0.60	0.61	0.63	0.66
Trp	0.18	0.18	0.18	0.20	0.20	0.17	0.19	0.20	0.20	0.22
Ile	0.64	0.65	0.67	0.71	0.71	0.63	0.64	0.68	0.70	0.74
Val	0.73	0.76	0.79	0.82	0.84	0.74	0.77	0.81	0.84	0.87
Total ash	12.5	12.4	12.6	12.9	12.6	12.8	12.9	12.7	13.2	12.9
Ether extract	5.70	5.95	5.80	6.12	6.21	6.75	7.06	7.01	7.52	7.41
Calculated analysis^4^										
AMEn (kcal/kg)	2,750	2,750	2,750	2,750	2,750	2,800	2,800	2,800	2,800	2,800
Linoleic acid	2.93	2.93	2.92	2.92	2.92	3.61	3.61	3.61	3.62	3.62
Crude fiber	4.66	4.53	4.40	4.28	4.15	4.64	4.52	4.40	4.27	4.15
DAA^5^										
Arg	0.95	0.99	1.02	1.05	1.08	0.97	1.01	1.04	1.07	1.11
Lys	0.66	0.69	0.72	0.75	0.78	0.67	0.70	0.73	0.76	0.79
Met	0.38	0.40	0.42	0.44	0.46	0.39	0.42	0.44	0.46	0.48
Met + Cys	0.59	0.62	0.64	0.67	0.69	0.60	0.63	0.66	0.68	0.71
Thr	0.48	0.49	0.51	0.53	0.55	0.48	0.50	0.52	0.54	0.56
Trp	0.15	0.16	0.17	0.17	0.18	0.16	0.16	0.17	0.18	0.18
Ile	0.54	0.57	0.59	0.61	0.64	0.55	0.58	0.60	0.63	0.65
Val	0.64	0.66	0.68	0.71	0.73	0.65	0.67	0.70	0.72	0.75
Calcium	3.78	3.78	3.78	3.78	3.78	3.86	3.86	3.86	3.86	3.86
Available phosphorus	0.33	0.33	0.33	0.33	0.33	0.34	0.34	0.34	0.34	0.34

1 Standardized ileal digestible lysine.

2 70% coarse (3 to 4 mm) and 30% fine (<0.6 mm).

3 Provided the following per kilogram of diet: vitamin A (*trans*-retinyl acetate), 8,000 IU; vitamin D_3_ (cholecalciferol), 3,000 IU; vitamin E (dl-α-tocopheryl acetate), 10 IU; vitamin K, 1 mg; vitamin B_1_, 1.0 mg; vitamin B_2_, 4 mg; vitamin B_6_, 1.5 mg; vitamin B_12_ (cyanocobalamin), 15 μg; niacin, 20 mg; pantothenic acid (d-calcium pantothenate), 8.2 mg; folic acid, 1 mg; biotin, 100 μg; choline (choline chloride), 200 mg; manganese (MnO), 70 mg; zinc (ZnO), 50 mg; iron (FeSO_4_.H_2_O), 30 mg; copper (CuSO_4_ 5H_2_O), 6 mg; iodine [Ca(IO_3_)2], 0.5 mg; selenium (Na_2_SeO_3_), 0.3 mg; Axtra PHY, 30mg [300 U of 4a24 6-phytase (EC 3.1.3.26)] supplied by IFF, Madrid, Spain.

4 According to FEDNA (2021).

5 Standardized ileal digestible amino acid.

### Laboratory Analysis

Representative samples of the diets were ground in a laboratory mill (Retsch Model Z-I, Stuttgart, Germany) equipped with a 0.75 mm screen and analyzed for moisture by oven-drying (method 930.15), gross energy using an adiabatic bomb calorimeter (model 6400, Parr Instrument Company, Moline, IL), total ash using a muffle furnace (method 942.05), and nitrogen by combustion (method 968.06) using a Leco analyzer (model FP-528, Leco Corp., St. Joseph, MI) as indicated by AOAC International (2005). Ether extract was analyzed after 3 N HCl acid hydrolysis (method 159 Am 5-04) as indicated by the AOCS (2017) using an Ankom XT10 extraction system (Ankom Technology Corp. Macedon, NY). The AA were analyzed by ion-exchange chromatography (Hewlett-Packard 1100, Waldbronn, Germany) as described by De Coca-Sinova et al. (2008). Briefly, representative samples of the diets were hydrolyzed in 6N HCl for 22 h at 110°C under reflux conditions. For the determination of Met and Cys, separate feed samples were oxidized with performic acid before hydrolysis, and measured as Met sulfone and cysteic acid, respectively. Tryptophan was determined after alkaline hydrolysis for 20 h at 110°C. All the analyses were conducted in duplicate.

### Measurements

#### Egg Production

All eggs produced were collected daily. Egg weight was measured in all the eggs laid the first day of each week of the 6 experimental periods (4 wk each) and the average value was used for further analyses. Feed disappearance and BW of the hens were determined by replicate and period. From these data, egg production, egg weight, egg mass, FI, and feed conversion ratio per kilogram of eggs (**FCR**) were determined by period (4 wk), phase (prepeak from 18 to 21 wk and peak from 22 to 41 wk of age), and cumulatively (18–41 wk of age). In addition, DLys intake, expressed in mg per day, energy intake, expressed as kcal AMEn ingested per hen per day, and energy conversion ratio (**ECR**), expressed as kcal AMEn/g of eggs, were calculated. Any mortality was recorded and weighed as it occurred. Finally, the number of days needed to reach 30% of egg production was measured to evaluate the effects of the diet on the initiation of the egg production cycle.

#### Egg Quality

The percentage of dirty, broken, and shell-less eggs was determined by 2 independent observers blind to treatment in all the eggs produced. An egg was considered as dirty when a spot of any kind or size was detected on the shell (Lázaro et al., 2003). Egg shell strength and albumen quality (Haugh Units) were measured in 8 fresh eggs collected randomly from each replicate for the last 2 days of the 6 experimental periods. The eggs were weighed individually and shell strength, expressed in g/cm^2^, was determined applying increased pressure to the broad pole of the egg, using an egg shell force gauge (Egg Force Reader, SANOVO Technology A/S, Odense, Denmark) as indicated by Safaa et al. (2008a). Haugh Units were measured using a multitester equipment (QCM System, Technical Services and Supplies, Dunnington, York, UK) as indicated by Pérez-Bonilla et al. (2012a).

### Statistical Analysis

Data were analyzed as a completely randomized design with 10 treatments arranged as a 2 × 5 factorial with AMEn and DLys to AMEn ratio of the diets as main effects, using the MIXED procedure of SAS (SAS Institute, 2018). Each treatment was replicated 10 times and the experimental unit was an enriched cage with 10 hens for all measurements. When the effects of the AMEn concentration or the DLys content of the diet on the different variables studied were significant, the Tukey test was used to separate treatment means. In addition, the effects of the DLys content on these variables, were partitioned into its linear and quadratic components and the data were analyzed using the regression procedure of SAS (SAS Institute, 2018). Age effect (6 periods of 4 wk each) and the interaction between age and main dietary effects (AMEn and DLys:AMEn ratio) on egg production and egg quality traits, were tested as indicated by Littell et al. (1998). Results in tables are presented as means and the differences observed were considered significant at *P* < 0.05.

## RESULTS

The determined chemical composition of the diets, including AA content, was in reasonable agreement with the calculated values (Table 1). Health status of the birds was good throughout the experiment. Mortality was low (2.3%) and was not related to any treatment (data not shown).

### Egg Production

Age affected significantly (*P* < 0.001) all egg production traits but no interactions between age and diet were detected. Similarly, no interactions between main effects of the diets were observed for any of the traits studied at any age and therefore, only main effects are presented.

### Prepeak Phase (18–21 wk of age)

#### Energy Concentration

From 18 to 21 wk of age, an increase in the AMEn content of the diet from 2,750 to 2,800 kcal/kg did not affect any of the variables studied except FI that was reduced by 3.2% in hens fed the high energy diets (84.4 vs. 87.1 g/d; *P* < 0.01). Energy intake, however, was not affected. Similarly, age at which the hens reached 30% egg production, was not affected by the energy content of the diet (Table 2).

**Table 2:** Influence of the AMEn (kcal/kg) and standardized ileal digestible lysine (DLys) contents of the diet on egg production from 18 to 21 wk of age (prepeak phase).

	AMEn (kcal/kg)		DLys^1^ (%)						*P*-value^3^^,^^4^		
								SEM^2^ (n = 10)	AMEn	DLys	
	2,750	2,800	0.665	0.695	0.725	0.755	0.785			L^5^	Q^5^
Egg production (%)	41.2	39.6	39.5	38.0	41.4	39.9	43.1	2.86	0.411	0.151	0.514
Days to 30% egg production	133.8	133.9	134.2	134.5	133.6	133.8	133.3	1.02	0.374	0.096	0.833
Feed intake (g/d)	87.1^a^	84.4^b^	85.9	85.3	85.7	86.4	87.2	1.38	0.003	0.086	0.201
Energy intake (kcal/d)	239	236	238	232	238	240	242	3.83	0.210	0.075	0.190
DLys intake (mg/d)	627	617	571^e^	580^d^	621^c^	652^b^	685^a^	10.4	0.093	<0.001	0.183
Egg weight (g)	50.3	50.5	50.2	49.8	51.0	50.4	50.6	0.77	0.733	0.427	0.820
Egg mass (g/d)	20.7	20.0	19.9	18.9	21.1	20.2	21.8	1.50	0.477	0.123	0.590
FCR^6^ (kg/kg)	4.385	4.431	4.603	4.680	4.158	4.418	4.182	0.4011	0.940	0.096	0.998
ECR^7^ (kcal/g of egg)	12.1	12.4	12.8	13.0	11.5	12.3	11.6	1.10	0.807	0.097	0.987
BW gain (g/d)	5.9	5.6	6.0	5.8	5.5	5.7	5.7	0.53	0.469	0.498	0.527

1 Values correspond to the average of the diets with 2,750 and 2,800 kcal AMEn/kg.

2 Standard error of the mean: 50 and 20 replicates for AMEn and DLys effects, respectively.

3 The interactions between main effects were not significant (*P* > 0.10) for all the variables studied.

4 Age effect was significant (*P* < 0.001) for all the variables studied.

5 Linear (L) and quadratic (Q) components.

6 Feed conversion ratio.

7 Energy conversion ratio.

a-e Within a line, means without a common superscript differed significantly (P < 0.05)

#### Standardized Digestible Lysine

An increase in the DLys:AMEn ratio from 0.24 to 0.28 mg DLys/kcal AMEn (equivalent to 0.66 to 0.78% and 0.67 to 0.79% DLys for the low and high AMEn diets, respectively) tended to increase linearly energy intake (*P* = 0.075) and ECR (*P* = 0.097) and to decrease the number of days needed by the hens to reach 30% egg production (*P* = 0.096).

### Peak Production Phase (22–41 wk of age)

#### Energy Concentration

An increase in the AMEn content of the diet of 50 kcal/kg, increased egg weight (60.7 vs. 59.9 g; *P* < 0.05) and tended to improve energy intake (304.6 vs. 308.9 kcal AMEn/d; *P* = 0.083) and FCR (2.027 vs. 2.002; *P* = 0.074). Egg mass production and ECR, however, were not affected (Table 3).

**Table 3:** Influence of the AMEn (kcal/kg) and standardized ileal digestible lysine (DLys) contents of the diet on egg production from 22 to 41 wk of age (peak production phase).

	AMEn (kcal/kg)		DLys^1^ (%)						*P*-value^3^^,^^4^		
								SEM^2^ (n = 10)	AMEn	DLys	
	2,750	2,800	0.665	0.695	0.725	0.755	0.785			L^5^	Q^5^
Egg production (%)	91.3	90.9	90,3^ab^	90,2^ab^	90,3^ab^	92,2^a^	92,4^a^	1.17	0.583	<0.001	0.165
Feed intake (g/d)	110.8	110.3	110,2	110,1	110,3	111,1	111,1	1.29	0.486	0.131	0.840
Energy intake (kcal/d)	304.6	308.9	305,7	305,4	306,2	308,2	308,2	3.58	0.083	0.131	0.845
DLys intake (mg/d)	798	806	733^e^	765^d^	800^c^	839^b^	872^a^	9.5	0.238	<0.001	0.807
Egg weight (g)	59.9^b^	60.7^a^	59.6^b^	60.1^b^	60.3^ab^	60.9^a^	60.8^a^	0.42	0.033	<0.001	0.194
Egg mass (g/d)	54.7	55.2	53,8^b^	54,2^b^	54,4^b^	56,2^a^	56,1^a^	0.75	0.342	<0.001	0.801
FCR^6^ (kg/kg)	2.027	2.002	2.050^a^	2.032^a^	2.030^a^	1.979^b^	1.981^b^	0.0232	0.074	<0.001	0.919
ECR^7^ (kcal/g of egg)	5.57	5.60	5.69^a^	5.64^a^	5.63^a^	5.49^b^	5.50^b^	0.06	0.495	<0.001	0.925
BW gain (g/d)	1.08	1.06	0,98	1,06	1,04	1,05	1,21	0.11	0.817	0.074	0.613

1 Values correspond to the average of the diets with 2,750 and 2,800 kcal AMEn/kg.

2 Standard error of the mean: 50 and 20 replicates for AMEn and DLys effects, respectively.

3 The interactions between main effects were not significant (*P* > 0.10) for all the variables studied.

4 Age effect was significant (*P* < 0.001) for all the variables studied.

5 Linear (L) and quadratic (Q) components.

6 Feed conversion ratio.

7 Energy conversion ratio.

a-e Within a line, means without a common superscript differed significantly (P < 0.05)

#### Standardized Digestible Lysine

An increase in the DLys content of the diet from 0.665 to 0.785% (average of the 2 set of diets differing in AMEn content) improved linearly egg production, egg weight, egg mass, FCR, and ECR (*P* < 0.001), and tended to increase BW gain (*P* = 0.074). Energy intake was not affected by the DLys content of the diet.

### Whole Experiment (18–41 wk of age)

#### Energy Concentration

**Figure 1:**
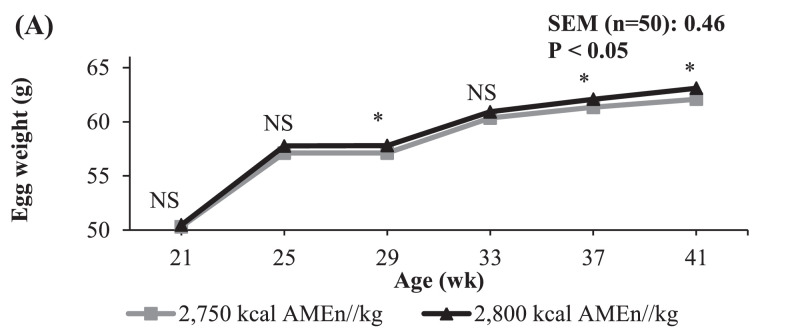
Influence of the AMEn content (kcal/kg) of the diet on egg weight from 18 to 41 wk of age^1^ (whole experimental period). ^1^Age effect (*P* < 0.001). ^NS^*P* > 0.05; *0.05 > *P* > 0.01.

An increase in the AMEn content of the diet of 50 kcal/kg did not affect any of the variables studied except egg weight that was 0.6 g heavier (59.2 vs. 58.6 g; *P* < 0.05) in hens fed the high energy diet (Table 4). Most of the benefits of the energy content of the diet on egg weight were observed during the last part of the experiment (Figure 1).

**Table 4:** Influence of the AMEn (kcal/kg) and standardized ileal digestible lysine (DLys) contents of the diet on egg production from 18 to 41 weeks of age (whole experimental period).

	AMEn (kcal/kg)		DLys^1^ (%)						*P*-value^3^^,^^4^		
								SEM^2^ (n = 10)	AMEn	DLys	
	2,750	2,800	0.665	0.695	0.725	0.755	0.785			L^5^	Q^5^
Egg production (%)	82.9	82.3	81.8	81.5	82.1	83.4	84.1	1.23	0.449	0.241	0.665
Feed intake (g/d)	106.7	105.8	106.0	105.5	106.0	106.8	106.9	1.21	0.234	0.266	0.747
Energy intake (kcal/d)	293.3	296.3	294.1	292.6	294.3	296.4	296.7	3.37	0.194	0.266	0.751
Egg weight (g)	58.6^b^	59.2^a^	58.3^b^	58.5^b^	58.8^ab^	59.4^a^	59.4^a^	0.51	0.045	0.013	0.449
DLys intake (mg/d)	768	773	705^e^	733^d^	769^c^	806^b^	839^a^	8.9	0.464	<0.001	0.221
Egg mass (g/d)	48.5	48.7	47.7	47.7	48.3	49.5	49.9	0.76	0.585	0.085	0.888
FCR^6^ (kg/kg)	2.201	2.176	2.227	2.214	2.200	2.158	2.145	0.0703	0.571	0.171	0.992
ECR^7^ (kcal/g of egg)	6.05	6.09	6.18	6.14	6.11	5.99	5.95	0.202	0.674	0.172	0.998
BW gain (g/d)	1.95	1.89	1.90	1.93	1.86	1.89	2.03	0.133	0.489	0.453	0.345

1 Values correspond to the average of the diets with 2,750 and 2,800 kcal AMEn/kg.

2 Standard error of the mean: 50 and 20 replicates for AMEn and DLys effects, respectively.

3 The interactions between main effects were not significant (*P* > 0.10) for all the variables studied.

4 Age effect was significant (*P* < 0.001) for all the variables studied.

5 Linear (L) and quadratic (Q) components.

6 Feed conversion ratio.

7 Energy conversion ratio.

a-e Within a line, means without a common superscript differed significantly (P < 0.05)

#### Standardized Digestible Lysine

**Figure 2:**
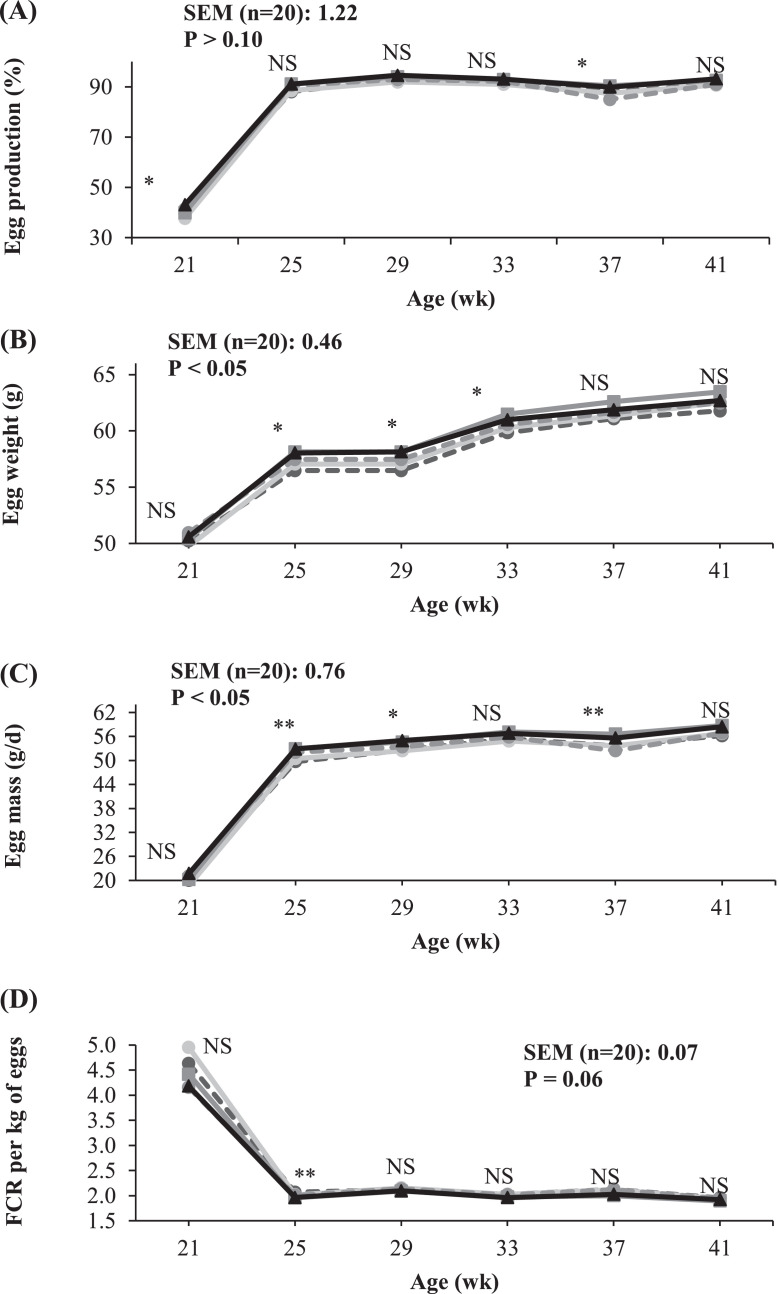
Influence of the standardized ileal digestible lysine (DLys) content of the diet on (A) egg production, (B) egg weight, (C) egg mass, and (D) feed conversion ratio (FCR) from 18 to 41 wk of age^1^ (whole experimental period). Only data from those variables showing significant effect for at least one experimental period, are reported. ^1^Age effect (*P* < 0.001). ^NS^*P* > 0.05; *0.05 > *P* > 0.01; **0.01 > *P* > 0.001.

An increase in the DLys content of the diet from 0.665 to 0.785% (average of the 2 set of diets differing in AMEn content) increased egg weight (*P* < 0.05) and tended to improve egg mass production (*P* = 0.085). Most of the benefits of the increase in DLys on these traits were observed from 25 to 37 wk of age (Figure 2).

### Egg Quality Traits

Age of the hens affected all egg quality traits studied (*P* < 0.001) but no interactions between age and diet were detected. Diet did not affect egg quality, except shell strength that increased linearly (*P* < 0.05) as the DLys content of the diet increased (Table 5).

**Table 5:** Influence of the AMEn (kcal/kg) and standardized ileal digestible lysine (DLys) contents of the diet on egg quality traits from 18 to 41 weeks of age (whole experimental period)

	AMEn (kcal/kg)		DLys^1^ (%)						*P*-value^3^^,^^4^		
								SEM^2^ (n = 10)	AMEn	DLys	
	2,750	2,800	0.665	0.695	0.725	0.755	0.785			L^5^	Q^5^
Dirty eggs (%)	0.45	0.37	0.41	0.42	0.39	0.43	0.39	0.099	0.389	0.554	0.909
Broken eggs (%)	0.12	0.09	0.08	0.11	0.11	0.14	0.09	0.053	0.204	0.426	0.321
Shell-less eggs (%)	1.54	1.48	1.53	1.44	1.46	1.63	1.50	0.188	0.646	0.972	0.984
Haugh units	97.6	97.8	97.4	97.5	97.9	97.7	98.1	0.39	0.960	0.835	0.981
Shell strength (kg/cm^2^)	4.271	4.266	4.225^b^	4.258^ab^	4.256^ab^	4.311^a^	4.294^ab^	0.038	0.815	0.026	0.664

1 Values correspond to the average of the diets with 2,750 and 2,800 kcal AMEn/kg.

2 Standard error of the mean: 50 and 20 replicates for AMEn and DLys effects, respectively.

3 The interactions between main effects were not significant (*P* > 0.10) for all the variables studied.

4 Age effect was significant (*P* < 0.001) for all the variables studied.

5 Linear (L) and quadratic (Q) components.

## DISCUSSION

No interactions between age and diet or between the main effects of the diet were detected for any of the traits studied and therefore, only main effects are discussed.

### Prepeak Phase (18–21 wk of age)

#### Energy Concentration

In general high energy diets are recommended in the prepeak phase to optimize egg production during the whole cycle (Hy-Line 2020; Isa Brown, 2020; Lohmann, 2020). In fact, Harms et al. (2000) and Pérez-Bonilla et al. (2012a) reported higher FI and dePersio et al. (2015) greater egg mass production in this phase, as the energy content of the diet increased. In the current research, however, an increase in the AMEn content of the prepeak diet from 2,750 to 2,800 kcal/kg did not affect energy intake or hen production, consistent with data of Leeson et al. (2001) and Saldaña et al. (2016). Similarly, Keshavarz (1998), Guzmán et al. (2016), and Scappaticcio et al. (2021) did not observe any effect of the energy content of the diet on these traits. The reasons for the discrepancies observed are not apparent but BW, age, and gastrointestinal tract development during the last phase of the rearing period, might have a greater effect on energy intake than an increase in the AMEn of diet of 50 kcal (Bouvarel et al., 2010; Leeson and Summers, 2012; Guzmán et al., 2015).

#### Standardized Digestible Lysine

An increase in DLys did not affect egg weight or egg production but tended to increase energy intake and to improve ECR. The limited effects of an increase in DLys content of the diet on egg mass production was expected because the DLys intake of the hens in this phase was above requirements. The DLys requirements of young hens are 100 mg per kg BW^0.75^ for maintenance, 20 mg per g of BW gain, and 12.4 mg per g of egg produced (Joly, 2012; Rostagno et al., 2017). In the current research, the average BW^0.75^, and BW gain, and egg mass production of the hens were 1.37 kg, 5.7 g/d, and less than 22 g/d, respectively, resulting in a total requirement of 505 mg DLys/d. On the other hand, average FI was over 85 g/d for all the groups, which corresponded to a minimum DLys intake of 571 mg/d.

Pullets fed the high DLys diets, reached 30% egg production one day sooner than pullets fed the low DLys diets, consistent with data of Kwakkel et al. (1991) who suggested that a deficiency in Lys affects the initial development of the reproductive tract. Kwakkel et al. (1995) reported that over 40% of the increase in BW gain of 19 wk-old hens, corresponded to the development of the reproductive organs. In fact, Silva et al. (2000) and Bunchasak and Silapasorn (2005) observed that most of the AA of the diet in young pullets (16–22 wk of age), were used for the development of tissues of the reproductive tract, which facilitated the initiation of the egg production cycle.

The data reported herein indicate that under the conditions of the current research, young hens required less than 571 mg DLys/d to optimize BW gain and egg production. However, if the objective is to promote an early start of the egg production (and probably better persistency during the egg production cycle), higher levels of DLys might be required (Macelline et al., 2021).

### Peak Production Phase (22–41 wk of age)

#### Energy Concentration

An increase in the AMEn content of the diet from 2,750 to 2,800 kcal/kg, improved energy intake, FCR, and egg weight but did not affect egg mass production, ECR, or BW gain. The increase in egg weight observed with an increase in dietary energy concentration, was consistent with data of Harms et al. (2000), dePersio et al. (2015), and Scappaticcio et al. (2021). In contrast, Wu et al. (2007) and Saldaña et al. (2016) did not find any increase in egg weight with increases in the energy content of the diet. The reasons for the discrepancy among authors, on the effects of the energy concentration of the diet on egg weight, are not apparent but depend probably, of the nutritional characteristics of the control diet (Safaa et al., 2008b; Herrera et al., 2018). In this respect, Pérez-Bonilla et al. (2012a) reported that an increase in energy was more beneficial when low rather than high energy diets were used, consistent with the results of the current research. On the other hand, under most practical situations, the level of supplemental fat increases as the energy content of the diet increases. It has been reported consistently, that the main reason for the increase in egg weight observed with the use of high energy diets (provided that the linoleic acid content was above 1.1%), was the increase in the level of supplemental fat (Grobas et al., 1999; Safaa et al., 2008b). In the current research, the linoleic acid content of all the experimental diets was over 2.9% and thus, the 0.8 g increase in egg weight observed could result from the extra fat included in the high energy diets. In fact, Safaa et al. (2008b) and Bouvarel et al. (2010) reported that per each 1% of extra supplemental fat, egg weight increased by 0.2 to 0.35 g.

#### Standardized Digestible Lysine

An increase in the DLys:AMEn ratio of the peak phase diets, corresponding to an increase in DLys from 0.66 to 0.78% and from 0.67 to 0.79% for the low and high energy diets, respectively, improved linearly egg weight, egg mass production, and FCR. Schutte and Smink (1998), Rocha et al. (2009), Matos et al. (2009), and Kumar et al. (2018) in white hens and Wijtten et al. (2006) and Scappaticcio et al. (2021) in brown hens, recommended a minimum intake of DLys of 720, 759, 676, 839, and 700 and 843 mg/d, respectively. Probably, differences in egg mass output and age of the hens at the start of the experiment, justify the variability in DLys requirements observed. The data reported herein confirm that brown hens producing over 56 g of egg/d require at least 839 mg DLys/d to maximize production, in agreement with data of Scappaticcio et al. (2021). Lemme et al. (2009) and Van Krimpen et al. (2015), conducted 2 meta-analytical studies with mixed strains of hens that included 19 and 6 experiments, respectively. The authors reported that the requirement in DLys to optimize hen production was of 810 and 855 mg/d, respectively, in agreement with the results of the current research. Feed conversion ratio improved linearly as the DLys content of the diet increased, consistent with data of Wijtten et al. (2006) and Spangler et al. (2018) with brown and white hens, respectively. Also, BW of the hens increased as the DLys content of the diet increased, in agreement with data of Wijtten et al. (2006) in brown hens and Rocha et al. (2009) in white hens of similar ages.

### Whole Experiment (18–41 wk of age)

#### Energy Concentration

The only variable affected by an increase of 50 kcal AMEn/kg diet was egg weight which increased with most of the benefits observed during the peak phase. Feed intake, was not affected during the whole experiment, in spite of the significant increase observed during the prepeak phase. Similar data have been reported by dePersio et al. (2015) who observed that an increase in the energy content of the diet of 200 kcal/kg (2,750–2,950 kcal/kg) had more relevant effects on FI of the hens from 19 to 26 wk than from 27 to 32 wk of age.

#### Standardized Digestible Lysine

An increase in DLys increased egg weight and egg mass production but did not affect any other production trait. In fact, the improvements observed occurred mostly during the peak production phase, an observation that was expected, because in the prepeak phase the DLys intake of the hens was above requirements.

### Egg Quality Traits

#### Energy Concentration

An increase in the AMEn content of the diet from 2,750 to 2,800 kcal/kg did not affect any egg quality trait during the entire experiment, in agreement with data of Grobas et al (1999), Wu et al., (2007), Valkonen et al. (2008), Saldaña et al. (2016), and Scappaticcio et al. (2021).

#### Standardized Digestible Lysine

An increase in DLys had limited effects on egg quality, in agreement with data of Bouvarel et al. (2010), Spangler et al. (2018), and Scappaticcio et al. (2021). In fact, the only trait affected was an increase in shell strength, consistent with data of Leeson and Caston (1997) and Keshavarz and Nakajima (1995). The shell matrix contains 70% protein (Simkiss and Taylor, 1957) and thus, low CP diets might not supply sufficient amounts of some non-essential AA, such as glycine, serine, and glutamic acid which are needed to optimize egg shell quality (Macelline et al., 2021). In this respect, Pereira et al. (2019) reported that glutamic acid increased the percentage of Ca in the shell, suggesting that this AA improved Ca excretion in the uterus and its fixation in the eggshell.

In summary, an increase in the energy content of the diet from 2,750 to 2,800 kcal AMEn/kg had limited effects on egg production from 18 to 21 wk of age but improved egg weight and FCR in the peak production phase. From 18 to 21 wk of age, a DLys intake of 571 mg/d, corresponding to the two diets with the lower percentage of DLys, was sufficient to maximize hen production, possibly because of the low egg mass production of the young hens in this phase. The data confirm that in the peak production phase (22–41 wk of age) brown egg-laying hens fed diets with 2,750 or 2,800 kcal AMEn/kg require at least 0.75 and 0.76% DLys, respectively, corresponding to an intake of 839 mg/d, to optimize egg mass production, egg weight, and feed efficiency.

## DISCLOSURES

The authors confirm that there are not conflicts of interest in this research.
